# Weight Management in a Patient With Smith-Magenis Syndrome: The Role of GLP-1 Receptor Agonists

**DOI:** 10.1210/jcemcr/luaf094

**Published:** 2025-05-29

**Authors:** Jorge César Correia, Timothy Frayling, Zoltan Pataky

**Affiliations:** Unit of Therapeutic Patient Education, WHO Collaborating Centre, Department of Primary Care Medicine, University Hospitals of Geneva, 1206 Geneva, Switzerland; Faculty Diabetes Centre, University of Geneva, 1206 Geneva, Switzerland; Faculty Diabetes Centre, University of Geneva, 1206 Geneva, Switzerland; Department of Genetic Medicine and Development, Faculty of Medicine, University of Geneva, 1206 Geneva, Switzerland; Unit of Therapeutic Patient Education, WHO Collaborating Centre, Department of Primary Care Medicine, University Hospitals of Geneva, 1206 Geneva, Switzerland; Faculty Diabetes Centre, University of Geneva, 1206 Geneva, Switzerland

**Keywords:** Smith-Magenis syndrome, obesity management, GLP-1 receptor agonists, behavioral impulsivity

## Abstract

Smith-Magenis syndrome (SMS) is a rare genetic disorder characterized by intellectual disability, behavioral challenges, sleep disturbances, and obesity. Managing obesity in SMS is complex due to the behavioral dysregulation. This case involves a patient with SMS who experienced significant weight gain from early in childhood, developing complications such as type 2 diabetes, dyslipidemia, and steatotic liver disease. Initial management with lifestyle changes was insufficient, leading to progressive weight gain. At age 18 years, subcutaneous semaglutide was introduced, resulting in marked improvements in impulsivity, food cravings, and weight control. However, because of a global shortage of this medication, at age 21 years, she was switched to the oral formulation of semaglutide, which led to a relapse in violent behavior, increased food intake, and weight regain. When subcutaneous semaglutide became available again, it was reinstated, stabilizing her weight and behavior. This case underscores the potential of glucagon-like peptide 1 receptor agonists (GLP-1 RAs) in managing both obesity and behavioral symptoms in SMS. While injectable GLP-1 RAs show promise, further research is needed to determine why they may be more effective than oral formulations. Further studies are needed to confirm the effectiveness of GLP-1 RAs and the dosage and explore alternative treatments for long-term obesity management in genetic syndromes.

## Introduction

Smith-Magenis syndrome (SMS) is a neurodevelopmental disorder caused in a majority of cases from a deletion of a portion of chromosome 17p11.2, which includes the retinoic acid-induced 1 (*RAI1*) gene, whereas a smaller proportion arises from disruptions affecting the *RAI1* gene itself [1, 2]. Both forms lead to a similar clinical presentation that may include intellectual disability, distinctive behavioral patterns, sleep disturbances, and a predisposition to obesity [1, 2]. Managing obesity in patients with SMS is especially challenging because of the complex interaction of behavioral issues, metabolic disturbances, and the pharmacological treatments often required to manage other symptoms [1-3].

Therapeutic patient education focusing on sustainable lifestyle changes is the cornerstone for the treatment of all cases of obesity [4, 5]. However, in cases of genetic forms of obesity, such as those associated with neurodevelopmental disorders like SMS, antiobesity medications play a particularly important role. They complement behavioral interventions by addressing the metabolic and hormonal factors contributing to weight gain.

While glucagon like peptide 1 receptor agonists (GLP-1 RAs) such as semaglutide have been shown to be effective in promoting weight loss in the general population [6], their application in syndromic obesity, such as that seen in SMS, is still an emerging area of study. This case study explores the role of semaglutide in managing both obesity and behavioral dysregulation in SMS, providing valuable insights for clinicians treating syndromic obesity.

## Case Presentation

The patient was referred to the neuropediatric unit for evaluation at age 8 years due to significant developmental and behavioral challenges, including speech delay, hyperactivity, and severe insomnia. Her medical history was notable for an unremarkable pregnancy and birth and no complications during the neonatal period. Developmentally, she began walking at age 2 years, and her parents noted a marked delay in speech, which prompted speech therapy.

By age 5 years, she presented behavioral issues including impulsivity, aggression, and difficulty interacting with peers. She was often described as restless and hyperactive, with frequent bouts of frustration. The patient exhibited phobic behaviors, especially fear of swimming pools and stairs. No neuropsychological evaluation had been performed by this point. Her sleep pattern was extremely disrupted, with frequent early morning awakenings despite treatment with risperidone.

Since childhood, her weight had consistently been over the 90th percentile. Pubertal development was within the normal range, with menarche occurring at age 12 years. Her fasting glucose levels remained normal until age 16 years, when impaired glucose tolerance was detected, progressing to type 2 diabetes at age 18 years, at which point she had grade I obesity with a body mass index (BMI) of 32.5 kg/m^2^, along with dyslipidemia and metabolic dysfunction-associated steatotic liver disease (MASLD). Given these complications, she was referred for a comprehensive weight management program, focusing on structured dietary management, physical activity psychological support, and pharmacological therapy.

## Diagnostic Assessment

Physical examination revealed typical dysmorphic features associated with SMS, including small hands, short 5th fingers, and bilateral syndactyly of the 2nd and 3rd digits. She also had trident-shaped teeth, a tented mouth, small feet, and a supernumerary nipple under her left clavicle.

Behaviorally, the patient exhibited low frustration tolerance, manifested by head-banging when she did not get her way. She also demonstrated hyperactivity and impulsivity, which were difficult to control. Despite early intervention with risperidone for sleep and impulsivity, her behavior remained challenging, with episodes of self-injurious behavior.

Laboratory testing at age 18 years confirmed type 2 diabetes, with a hemoglobin A1c of 7.8% (normal reference range 3.0%-6.0%), leading to the initiation of metformin for glycemic control. Lipid profiles showed low high-density lipoprotein-cholesterol and mild hypertriglyceridemia. Liver function tests revealed a mild transaminitis, and abdominal ultrasound confirmed mild hepatic steatosis, supporting the diagnosis of MASLD.

No significant cardiac anomalies were noted in previous evaluations, and routine echocardiography had shown no abnormalities. Genetic testing confirmed the diagnosis of SMS, identifying a 17p11.2 deletion.

## Treatment

Management of the patient's body weight and metabolic complications required a multidisciplinary approach involving dietary modifications, physical activity promotion, pharmacological interventions, and psychological support, including cognitive-behavioral therapy. Initially, she was treated with metformin 500 mg twice daily to address her insulin resistance and type 2 diabetes. Despite dietary efforts and the introduction of regular physical activity (2 hours of school-based exercise per week), the patient's weight remained elevated, and her BMI persisted in the obesity grade I range.

**Figure 1.**
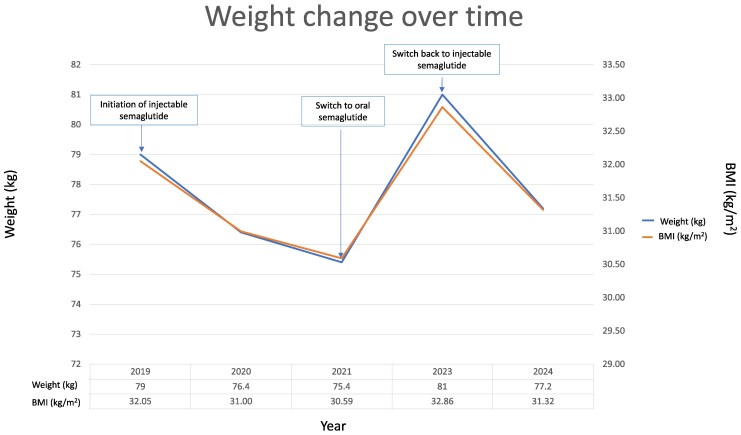
Weight change over time. Weight and BMI evolution of the patient following treatment with injectable and oral semaglutide. In 2019, subcutaneous semaglutide was introduced at a weight of 79 kg, resulting in a steady decline over 4 years, reaching 76.4 kg (BMI 30.99 kg/m^2^) in 2020 and 75.4 kg (BMI 30.58 kg/m^2^) in 2021. Her weight remained stable until 2023, when the patient was switched to oral semaglutide; this was associated with increased weight to 81 kg (BMI 32.86 kg/m^2^). Upon reintroduction of injectable semaglutide in 2024, weight decreased to 77.2 kg (BM 31.31 kg/m^2^) and stayed stable. BMI, body mass index.

In 2019, at age 18 years, the patient was started on subcutaneous semaglutide, a GLP-1 RAs, at the dosage of 0.25 mg weekly, gradually titrated to 1 mg weekly. This treatment was introduced to help manage not only her obesity and diabetes but also the behavioral challenges. The effects of semaglutide were notable; she started to progressively lose weight, from an initial weight of 79 kg in 2019 reaching 76.4 kg in 2020 and 75.4 kg in 2021 (Fig. 1) and her impulsivity and food cravings significantly decreased. Additionally, the patient and her family reported improvements in her mood, social interactions, and overall emotional regulation, which had a profound impact on her quality of life. According to the parents, for the first time, their daughter's mood has improved significantly, with reduced irritability and aggression.

Her weight remained stable until 2023, still aged 21 years going on 22, when due to a global shortage of subcutaneous semaglutide, she was switched to the oral formulation at the dosage of 14 mg once daily. Unfortunately, the oral form did not produce the same behavioral or metabolic improvements. Within a few weeks, the patient exhibited a relapse of violent behavior, increased food intake, and a resurgence of anxiety. These symptoms led to worsening social interactions, particularly in school, where she became more impulsive and aggressive. Her weight also increased to 81 kg (+4.6 kg) within a year, reversing the progress made on the injectable form despite continued efforts to maintain dietary control (Fig. 1).

In January 2024, at age 22 years, with the reavailability of subcutaneous semaglutide, this treatment was reinstated. She started losing weight again reaching, 78.3 kg over the following year, and her behavioral symptoms improved, with a marked reduction in impulsivity and food cravings.

## Outcome and Follow-up

The patient has been regularly followed up for 5 years, with her most recent evaluation in September 2024. Since resuming injectable semaglutide in January 2024, her weight has remained stable at 77.2 kg, with no further significant increases. Her type 2 diabetes remains well-controlled, and her hemoglobin A1c is now 6.5% (normal reference range 3.0-6.0%). Liver function tests continue to show mild transaminitis, and abdominal ultrasound show a stability of the steatosis, but her overall metabolic profile has improved, with normalized high-density lipoprotein-cholesterol and triglyceride levels.

Behaviorally, the patient has demonstrated significant improvement. As reported by her parents, her impulsivity is better controlled, and her social interactions, particularly at school, have improved markedly. She has become more engaged in social activities and she reports feeling more emotionally stable.

No significant adverse events have been reported with injectable semaglutide. The patient and her family are satisfied with the current management plan, and follow-up is scheduled every 3 months to monitor her body weight, metabolic health, and behavioral progress.

## Discussion

Obesity in SMS arises from a combination of genetic, behavioral, and neuroendocrine factors. The *RAI1* gene haploinsufficiency, which underlies SMS, disrupts regulatory pathways involved in appetite and energy balance, particularly affecting brain-derived neurotrophic factor expression in the hypothalamus [7, 8]. This disruption contributes to hyperphagia, reduced energy expenditure, and altered satiety signaling [7, 8]. Additionally, behavioral characteristics of SMS, including compulsive eating and food-seeking behaviors, further promote weight gain [9]. Neuroendocrine dysfunction also plays a role, particularly through dysregulation of the melanocortin-4 receptor (MC4R) pathway, which is central to appetite control and energy homeostasis [7, 8]. Although specific MC4R mutations are not typically associated with SMS, altered functioning of this pathway may contribute to the increased obesity risk observed in affected individuals. These combined factors make weight management in SMS particularly challenging and necessitate targeted interventions.

Patients with SMS appear to develop type 2 diabetes, MASLD, and dyslipidemia at a lower BMI compared to the general population, also due to the combination of genetic, metabolic, and neuroendocrine dysfunctions [3]. The *RAI1* gene has also been implicated in glucose and lipid metabolism, potentially predisposing individuals to insulin resistance even in the absence of severe obesity [3, 10]. Additionally, chronic hyperphagia and altered energy balance contribute to persistent metabolic stress, exacerbating insulin resistance and increasing the risk of diabetes. Neuroendocrine dysfunction, particularly involving the MC4R pathway, may also impair metabolic homeostasis, leading to dyslipidemia and an increased propensity for hepatic steatosis [7]. Furthermore, circadian rhythm disturbances, a hallmark of SMS due to the inversion of melatonin secretion, may further disrupt glucose metabolism and promote hepatic fat accumulation [11, 12]. Together, these factors create a state of metabolic vulnerability, where even modest weight gain can accelerate the onset of metabolic diseases, making early screening and intervention crucial in this population.

Medications are not the first line of treatment for obesity in SMS but should be considered in cases where behavioral interventions are not sufficient. Although the use of GLP-1 RAs is increasingly recognized in the management of obesity, there are no guidelines to their use specific to the treatment of obesity in syndromic conditions like SMS.

Although there are no SMS-specific studies on GLP-1 RAs, Prader-Willi Syndrome (PWS), which shares behavioral and metabolic features with SMS, has been the subject of several studies. For instance, exenatide, liraglutide, and semaglutide have been shown to reduce BMI, food intake, and hyperphagia in PWS, offering insights into their potential efficacy in syndromic obesity [13, 14]. Like SMS, patients with PWS benefit from medications that modulate appetite and impulsivity, making GLP-1 RAs a promising option for weight and behavior management in both syndromes.

In this case, injectable semaglutide demonstrated a notable improvement in both weight control and behavioral symptoms, particularly reducing impulsivity and food cravings. GLP-1 RAs have been shown to influence cognitive and behavioral regulation, potentially explaining their effects in this SMS case. Their impact is largely mediated through GLP-1 receptors in key brain regions such as the prefrontal cortex, nucleus accumbens, amygdala, and hypothalamus, which govern reward processing, impulse control, and emotional regulation [7, 15]. By modulating these pathways, GLP-1 RAs reduce compulsive behaviors, decrease hyperphagia, and improve emotional stability, which was observed in this patient when she was treated with injectable semaglutide. Additionally, GLP-1 activation affects the dopaminergic reward system, diminishing excessive food-seeking behaviors and impulsivity, contributing to the improvements in behavior and social interactions noted in this case [16]. The neuroprotective and anti-inflammatory effects of GLP-1 further support its role in cognitive stabilization by reducing oxidative stress and neuroinflammation, which are implicated in behavioral dysregulation [17].

The difference in effectiveness between injectable and oral semaglutide in this case likely reflects differences in bioavailability and central nervous system penetration, as injectable formulations achieve more consistent receptor activation [18]. When the patient was switched to oral semaglutide, behavioral symptoms worsened, with increased anxiety, aggression, and hyperphagia, suggesting diminished neurological effects.

Studies have found that to be the case, with subcutaneous semaglutide reducing body weight and blood sugar levels significantly more than the oral formulation [18]. However, it could also be a question of dosage, as current studies have shown promising results in terms of weight loss with oral semaglutide up to 50 mg daily [19]. Evidence is lacking regarding the behavioral impact the different formulations might have.

Setmelanotide, an MC4R agonist, is another treatment being evaluated for the treatment of syndromic obesities [20-22]. It has shown interesting results in reducing body weight in patients with Bardet-Biedl syndrome [22] but inconclusive results in Alström syndrome [22] and PWS [23]. One study assessed its effectiveness in SMS and the results showed no significant reduction in body weight [7].

Several limitations impacted the management of this case including the lack of standardized treatment protocols for SMS-specific obesity. Therefore we relied on general guidelines for obesity and diabetes management. More syndrome-specific guidelines are needed to better address the unique challenges of SMS.

## Learning Points

- The integration of a comprehensive patient education program, involving dietary, psychological, physical activity, and pharmacological interventions is key for weight management and improving overall health outcomes in patients with SMS
- GLP-1 receptor agonists, particularly the injectable formulation, can have a dual benefit in SMS, not only helping with weight control but also significantly improving impulsivity and behavioral stability, which are common challenges in SMS.
- Although GLP-1 receptor agonists like semaglutide show potential for treating SMS-related obesity and behavior, the variability in response between different forms (injectable vs oral and optimal dosage) indicates the need for further research to optimize treatment protocols and better understand their effects in genetic obesity syndromes.

## Contributors

J.C. and Z.P. are involved in the diagnosis and management of the patient. J.C. drafted the first draft of the manuscript. J.C., T.F., and Z.P. made individual contributions to the manuscript. All authors reviewed and approved the final draft.

## Data Availability

The authors confirm that the data supporting the findings of this study are available within the article.
